# The difference in dose and image quality between magnification methods used after the introduction of larger 60-inch operator screens

**DOI:** 10.1259/bjro.20190044

**Published:** 2020-04-21

**Authors:** Hywel Mortimer-Roberts, Michael R Rees

**Affiliations:** 1 North Wales Cardiac Centre, Betsi Cadwaladr University Health Board, Sarn Lan, United Kingdom

## Abstract

**Objective:**

To determine whether the use of display matrix magnification on larger operator screens without the use of conventional magnification can reduce radiation dose to the patient, and what effect it would have on image quality.

**Methods:**

The kerma-area product (KAP) resulting from standard projections in cardiac angiography were measured when an anthropomorphic phantom was imaged using conventional magnification method and display matrix magnification. The image quality was also evaluated by three observers using a TOR 18FG test tool for both magnification method.

**Results:**

The mean radiation KAP for the seven views with conventional magnification was 36.65 µGy m^−2^ whilst a reduction in KAP of 20.4% is possible using display matrix magnification (*p* < 0.05). The image resolution during acquisition was identical between both methods and only slightly reduced for the display matrix (1.6 LP mm^−1^) compared to conventional magnification (1.8 LP mm^−1^) when images were stored and retrieved on a Picture Archiving and Communication Systems (PACS) system. Both methods retained the same low-contrast detectability to PACS, with only a slight increase in detectability of 18 for display matrix magnification compared to 17 for conventional.

**Conclusion:**

Using display matrix magnification instead of conventional equipment magnification significantly reduces radiation does in all standard cardiac views without reducing image quality for the operator. This reduction in radiation dose is significant (*p* < 0.05) for the patients. The resolution did not change during acquisition, but contrast improved slightly (0.9% threshold contrast), but lost resolution of 0.2 LP mm^−1^ when archived to PACS.

**Advances in knowledge:**

This is a new method of reducing significant dose to the patient during cardiology examinations and may encourage further studies in other fluoroscopy lead examination to see if it could work for them.

## Introduction

During a new installation in a cardiology interventional department that opted for a large 60-inch operator monitor, it became apparent that the large monitor could be used to reduce the need for conventional magnification during cardiac examinations. Some operators adopted this new method of magnification (display matrix magnification) quickly, where others were sceptical, believing that the image was of reduced quality. They also believed that when archived, the image resolution was significantly reduced compared to conventional magnification methods, believing the resultant dose saving was not justifiable. Doubting this to be correct, combined with a desire to reduce radiation dose to the patient and the operator during procedures was the stimulus to this research.

The latest guidelines from the American College of Cardiology highlighted how the cardiologist and radiographer could reduce X-ray dose to the patient by using a lower frame rate, collimating the image and reducing exposure time.^1,2^ However, there is little emphasis regarding equipment adaptations such as larger monitors, unlike Gailloud^3^ who stated that larger monitors might have a role in dose reduction.

The use of conventional magnification while using image intensifiers cause significant dose increase during cardiology examinations. This increase in dose is due to an automated system inside the X-ray unit (the Automatic Brightness/Automatic Dose Rate Control System), designed to alter the X-ray factors when changes occur to the area examined.^4^ During magnification, the system increases the exposures, and therefore the dose to the patient, to maintain image quality.^5^ Flat-panel detectors, on the other hand, still result in dose increase but are less significant.^6,7^

Fluoroscopy equipment with a digital detector magnifies the image by exposing a smaller area of the detector and increase the size of the resultant image on the display screen. In theory, this should not result in any dose increase to the patient. However, as this process reduced the number of X-rays coming from the tube, its effect will reduce the X-ray beam intensity, requiring more exposure to maintain the same level of image quality, and reduce noise on the resultant image.^8^

Interventional cardiology procedures are becoming more advanced and more complex, often resulting in very prolonged radiation exposure times and consequent doses to patients and operators.^9^ Cardiology departments should always consider the latest in equipment design when updating or commissioning their cardiac catheterisation laboratories, in order to ensure they have optimised equipment designed for both patient and operator-specific issues and concerns.^2^

The option to choose a larger display during the installation of a new fluoroscopy unit will allow another method of magnification compared to a system that has been installed with small conventional monitors. The software running the larger displays can divide the screen into several regions (display matrix), each region may display any input the system is attached to, such as the live view, reference image, electrocardiogram, or intravascular ultrasound. This software now allows the live image to be displayed over a larger area of the screen, effectively magnifying the image (display matrix magnification).^3^ Display matrix magnification could replace conventional magnification techniques as a means of achieving diagnostic imaging without the consequent dose increase resulting from conventional equipment magnification.

Imaging archives (PACS, Picture Archiving and Communication Systems) are designed to keep, transfer, and display images from previous examinations over a more extended period than the X-ray equipment could hold. The size of the archive should reflect the workload of the department, how long they wish to store their images, and their underlying budget.^10,11^ Therefore, it is essential not to fill the archive with images that are not diagnostic. Images retrieved from an archive may be of lower resolution compared to the images from the original acquisition in order to save space.^12^ Therefore, it is critical to determine if any change to the magnification method would impact the archived images, in order to ensure the images are suitable for later review and planning of future treatment.^13^

This investigation was carried out in order to determine whether the use of display matrix magnification can reduce X-ray dose to the patient compared to conventional magnification. It was also essential to investigate the image quality during and after image acquisition, and archival, to ascertain if the method reduces dose while retaining excellent image quality.

The objectives of the study, therefore, were to:

1. Determine the difference in dose to a simulated anthropomorphic phantom for both magnification methods
2. Ascertain the difference in the image quality of both magnification methods during a simulated cardiac procedure and after archival.

## Limitations and conflict of interest

All individuals involved in the study acknowledge no known conflict of interest and have not received gifts or enumeration for the study and its contents. As the motivation for the study was made due to the installation of a large 60-inch monitor after an older system was replaced, the study was only performed on one manufacturer's equipment. For the study to have assessed further manufacturers equipment, it would have required offsite study, on equipment that also had the same large monitor option, and software comparable to perform display matrix magnification.

## Methods and materials

### Materials

The fluoroscopy unit used in this investigation was the Siemens Artis Zee, which uses conventional magnification factors of 25, 20, 16, and 10 cm, and was installed with a 60-inch operator display. Siemens uses zoom dose factors to describe the resultant dose for each magnification method. It is standardised across their range of detector sizes, and the factors for the equipment used for this research were as follows^14^ :

Zoom 0 - 25 cm pixels 960 × 960 zoom dose factor 83%.

Zoom 1 - 20 cm pixels 776 × 776 zoom dose factor 110%

Clinically, it is rare for the cardiologist in the department to use a higher magnification factor than “zoom 1” during their diagnostic and interventional procedures. Therefore, higher magnifications were not examined in this project. Siemens^14^ data suggest the dose would reduce by 27% if images are done without conventional magnification. However, as the Automatic Brightness/ Dose Rate Control system contributes to this change in dose by altering exposure factors, it was important to evaluate actual dose reduction values using both magnification methods.

The equipment was programmed to maintain the collimation and filter position regardless of X-ray tube position. By locking these, it allowed collimation and filter position to become a controlled variable. The Siemens Artis Zee's automated system can change more than just the exposure factors (such as output filter and pulse width). As this study was designed to be a simulated adult study, any of these changes would also alter during a genuine case, and the resultant change in dose, due to magnification method, could be used to evaluate this difference.

The X-ray equipment automatically records dose as a kerma-area product (KAP). This integrated dosemeter is calibrated annually and has bimonthly quality assurance checks. When the patient height and weight are entered, the equipment also calculates reference air kerma (RAK) as skin dose.^15,16^ This calculated skin dose was used in a previous study^3^ to ascertain dose reduction using display matrix magnification. However, as this value is based on patient height and weight, and only designed by the manufacturers as a guide to skin dose, it was felt more accurate to recorded only KAP^17^ as the value will not have been manipulated.

Additional equipment included an anthropomorphic phantom (Lungman Chest Phantom N1) to represent an adult patient as it absorbs X-rays in the same way as human tissues. ^18^ There is no simulated height and weight to the phantom, therefore, the equipment cannot calculate skin dose accurately, and cannot be considered an accurate dose recording method. A departmental test object MR000355 was created to allow accurate 10 × 10 cm square collimation, and the TOR 18FG test tool was used to determine the differences in image quality between both magnification methods.^19^

### Methodology

The X-ray equipment was positioned to ensure the phantom's^18^ heart was in the centre of the field of view and positioned to represent a patient during an examination. Doing so would allow the phantom to remain stationary throughout the examination without the need to reposition. The only movement would be from the fluoroscopy c-arm. By setting the tube hight to 105 cm, it allowed the tube to move to each position without colliding with the phantom, restricting tube angulation as the only movement variable.

A limit of 4 s was set on the fluoroscopy console automatically terminating the exposure electronically once this time has elapsed. Although this time may be too low to stabilise the signal, it is comparable to an actual examination where the operator would not wait for the signal to stabilise before the acquisition takes place as this would result in unwanted dose to the patient. Locking the exposure time would remove human bias or error and allow acquisitions to be comparable. The fluoroscopy system also records the number of frames acquired during those 4 s and was used as another method of ensuring the acquisitions were consistent.

The collimation test object (MR000355) was used to collimate the X-ray beam to exactly 10 × 10 cm, and it ensured the consistency of this variable during both phases of the study. Similarly, the fluoroscopy filter was positioned to cover the angle of the heart made up of the silhouettes of the left atrium, ventricles and lungs.

A simulated, seven projection investigation of the left coronary vessels was performed (see results section for actual tube angulations). The dose (KAP) for each projection was recorded using both magnification methods before the tube was moved to the next simulated position. This approach was used to ensure there was no difference to the anatomical or equipment position between the two acquisitions other than the magnification method used.

Using the department's quality assurance protocol for measuring image quality (low-contrast detectability and resultant resolution) of the acquired images, the TOR 18FG was used to identify the image quality of both magnification methods on the 60-inch operator screen at a distance of 1 m. The object contains a range of 18 contrast disks that are counted to indicate how well the system displays low-contrast (between 16.7 and 0.9% threshold contrast). Also contained in the test object is a range of 21 lined squares that are counted until the lines are no longer visible indicating the resolution of the system (between 5 and 0.5 line pairs per millimetre).^19^

The archived images were also evaluated on the large monitor to ensure that any change in image quality was due to image compression, rather than a different monitor. Two cardiologists were also asked to evaluate the resultant images using the quality assurance protocol privately and without discussion, and the results of all three observers were averaged to ensure subjectivity was kept to a minimum.

## Results

Tables 1 and 2 were created using the exposure reports generated by the X-ray equipment, and Table 3 was created by averaging the results from the three observers mentioned previously.

**Table 1.** KAP dose as a result of display matrix magnification

Acquisition number	LAO/RAO	CAU/CRA	kV	mA	ms	Focus	Dose µGym^2^	# of frames
2	PA	PA	81	230	3.5	Small	14.16	61
4	RAO 30°	CAU 30°	81	252	3.4	Small	15.35	61
6	PA	CAU 30°	81	350	5.1	Small	30.75	61
8	LAO 30°	CAU 40°	100	277	9.4	Small	75.12	61
10	LAO 30°	CRAN 30°	81	348	4.9	Small	29.36	61
12	PA	CRAN 40°	81	349	4.9	Small	29.60	61
14	RAO 30°	CRAN 30°	81	155	3.5	Small	9.76	61
						TOTAL	204.1	

CAU, Caudal; Cran, Cranial; Gy, Gray; KAP, kerma-air product; LAO, left anterior oblique; PA, Posteroanterior; RAO, right anterior oblique;kV, kilovoltage; mA, milliamperes; ms, milliseconds.

**Table 2.** KAP dose as a result of conventional magnification

Acquisition number	LAO/RAO	CAU/CRA	kV	mA	ms	Focus	Dose µGym^2^	# of frames
3	PA	PA	81	320	3.4	Small	19.12	61
5	RAO 30°	CAU 30°	81	332	3.5	Small	20.08	61
7	PA	CAU 30°	81	349	6.7	Small	40.92	61
9	LAO 30°	CAU 40°	109	254	9.4	Small	83.58	61
11	LAO 30°	CRAN 30°	81	351	6.7	Small	40.16	61
13	PA	CRAN 40°	81	352	6.6	Small	39.84	61
15	RAO 30°	CRAN 30°	81	208	3.5	Small	12.88	61
						TOTAL	256.58	

CAU, Caudal; Cran, Cranial; Gy, Gray; KAP, kerma-air product; LAO, ; PA, ; RAO, ;kV, kilovoltage; mA, milliamperes; ms, milliseconds.

**Table 3.** Image quality comparison between acquisition and PACS storage images as a result of magnification method

	Acquisition		PACS	
	Low-contrast details	Resolution LP mm^−1^	Low-contrast details	Resolution LP mm^−1^
Conventional magnification	17	2.5	17	1.8
Display matrix magnification	18	2.5	18	1.6

LP, Line Pairs;PACS, Picture Archiving and Communication System.

An average KAP reading of 36.65 µGym^2^ was measured during the seven exposures using conventional magnification. While using display matric magnification, a 20.4% reduction in KAP is possible (*p* < 0.05). The sample was unlikely to be from a normal distribution, and a Wilcoxon's signed-ranks (matched pairs) test was used as a nonparametric method of a significance test. The two-sided p-score was found to be 0.0156 with a 95.3% confidence interval for differences in KAP between both magnification methods.

The three observers showed perfect agreement (κ coefficient 1.00)^20^ when it came to image quality, scoring the same result for resolution and contrast detectability for each of the images. In terms of significance, due to the sample size and distribution, the data were inadequate for the Mann–Whitney *U* or Wilcoxon Signed Rank tests. Parametric tests were also inappropriate as normal distribution could not be expected or determined.

During acquisition conventional magnification showed 17 contrast disks (1.1% threshold contrast) and a resolution of 2.5 line pairs per millimetre, during display matric magnification, the resolution did not change, however, contrast improved to 18 contrast disks (0.9% threshold contrast). On exporting the images to PACS, the contrast levels did not change. However, the resolution for conventional magnification images sent to PACS was 1.8 LP mm^−1^ and did reduce to 1.6 LP mm^−1^ for display matrix resolution.

## Discussion

Several methods have been proposed to reduce radiation dose to the patient. Many of these are referred to in the latest multisociety consensus guidelines produced in 2018.^1^ The only methods used to address the issue of radiation scatter and absorbed dose are the conventional dose reduction measures of exposure values, exposure time, collimation, distance, and shielding. Guilloud^3^ earlier stated that larger monitors might be used to improve dose reduction, rather than be a simple hardware choice during purchasing.

Significant skin and other forms of radiation-induced injury may result from high examination doses,^21^ therefore a 20.5% reduction in dose of this significance (*p* < 0.05) would be classed as another essential method of dose reduction to those stated previously.^1^ Including Guilloud,^3^ as this study demonstrated a significant dose (KAP) reduction in dose, manufacturers may refer to the larger monitors and matrix displays as more than a simple aesthetic or operator preference.

Using the information supplied by Siemens,^14^ it could be calculated that the difference in dose between the magnification levels studied should have resulted in a 27% approximate reduction in dose. The results show that the actual dose reduction using display matrix magnification was approximately 20.5%. As the results show, the automatic brightness/dose rate control system did alter the exposure factors contributing to some of the dose reduction. The calculations performed by Siemens^14^ may not be able to predict these changes, though a lower dose reduction was achieved, it was still a significant reduction (*p* < 0.05).

As an additional finding of this study, specific projections have a higher radiation dose than others. A reduction in the use of a combination of caudal and left anterior oblique angulations may also significantly reduce the dose to the patient.^22^ With the reduction in dose of 20.5% to those projections, it may allow a cardiologist to continue working in those projections for a greater length of time, which may be beneficial to the procedure if that view is the only projection to show the lesion adequately.

Due partly to the automatic brightness/dose rate control system, the image quality will change due to changes in exposure factors. However, as this study has attempted to simulate a cardiac procedure, it is important to note how the magnification method altered the image quality. There was a small increase in low-contrast detectability using display matrix magnification of 18 (0.9% threshold contrast) compared to 17 (1.1% threshold contrast) for conventional magnification. It is important to note that during both methods, the system maintained the same 81 kV. Therefore, it is unsurprising that the contrast did not alter significantly (0.2% threshold contrast), but was in favour of display matrix magnification.

In terms of resolution, the results show that during the procedure, there is no discernible difference in resolution between magnification methods. During compression of the images to archive, the resolution drops from 1.8 LP mm^−1^ for conventional magnification to 1.6 LP mm^−1^ for display matrix magnification. The slight difference in resolution means that an object of 0.2 mm or less may become unnoticeable compared to the conventional magnification method, but only for the archived images. The loss of resolution by different types of compression in PACs systems has been studied extensively.^23^ Reluctance to use the new method of magnification by the operators initially may have been due to how different the images appeared on the operator screen. This research shows that the resolution of the images is identical. Therefore, it may merely require the operators to become accustomed to the new image appearance while being aware of the benefit in dose reduction to them and the patient.

A stationary test object was used to acquire the resolution results in this study and not a dynamically moving structure. Therefore, there may be differences in visualising structures of a dynamically moving heart compared to a stationary test object. However, as the resultant resolution difference between both methods of magnification was so small when sent to PACs. As there was no difference in resolution of the original images, it may be suitable to accept the dose reduction benefit for the patient compared to the very minimal loss in resolution of the archived images. This issue could be addressed by purchasing more storage space on in the PACS archives or compensated for by the level of compression used.

As stated by the IAEA,^9^ the dose reduction techniques they suggested could also reduce the risk of radiation exposure to the operator and other staff in the cardiac theatre. Therefore, the findings of this study would imply a potential dose reduction to staff as well as the patient. It may be appropriate to follow up this study with a retrospective dose assessment of operator dosemeters for their hands, torso, and eye lenses, in the months before and after the introduction of the new magnification method. The department records all patients height and weight and total dose (KAP), and the operators have monthly recorded hand, torso and eye lenses doses. It may be possible to compare several months worth of data before and after the adoption of this magnification method and compare the changes in dose to the operator and patient under a range of different patient demographics and procedure type.

## Conclusion

This study has demonstrated that there is a significant 20.4% dose reduction (*p* < 0.05) possible for patients undergoing fluoroscopy-guided cardiac procedures using display matrix magnification on a large 60-inch display compared to conventional X-ray tube magnification. Compared to conventional magnification, display matrix magnification showed no change in resolution of the image and only a slight increase in discernible low-contrast detail during acquisition (0.2% threshold contrast). Once the acquisition images were sent to PACS, display matrix magnification showed no difference in low-contrast detail, but a slight decrease in resolution of 0.2 LP mm^−1^.

## References

[1] Hirshfeld JW, Ferrari VA, Bengel FM, Bergersen L, Chambers CE, Einstein AJ, et al 2018 ACC/HRS/NASCI/SCAI/SCCT Expert Consensus Document on Optimal Use of Ionizing Radiation in Cardiovascular Imaging—Best Practices for Safety and Effectiveness, Part 2: Radiological Equipment Operation, Dose-Sparing Methodologies, Patient and Medical Personnel Protection. J Am Coll Cardiol 2018; 71: 2829–55. doi: 10.1016/j.jacc.2018.02.018 29729878

[2] ICRP Radiological protection in cardiology. ICRP publication 120. ICRP 2013; 42. 10.1016/j.icrp.2012.09.001 23141687

[3] Gailloud P A large display is a powerful tool to reduce radiation exposure during single-plane fluoroscopically guided procedures. AJR Am J Roentgenol 2015; 204: W483–5. doi: 10.2214/AJR.14.13246 25794099

[4] AAPM Task Group 125 Functionality & Operation of Fluoroscopic Automatic Brightness Control/Automatic Dose Rate Control Logic in Modern Cardiovascular & Interventional Angiography Systems Report No.: 125 College Park MD: American Association of Physicists in Medicine, Science Council;. 2012. 10.1118/1.4704524 22559654

[5] International atomic energy Agency. good practices in fluroscopy 2019;Available from.

[6] Nickoloff EL AAPM/RSNA physics tutorial for residents: physics of flat-panel fluoroscopy systems: survey of modern fluoroscopy imaging: flat-panel detectors versus image intensifiers and more. Radiographics 2011; 31: 591–602. doi: 10.1148/rg.312105185 21415199

[7] Huang SY, Jones K Image Wisely. [Online]. 2014 Available from: http://www.imagewisely.org/imaging-modalities/fluoroscopy/articles/huang-patient-specific-factors. [cited 2017 September 4].

[8] Fauber TL 5th ed Radiographic Imaging & Exposure: St. Louis: Elsevier; 2017.

[9] International atomic energy Agency Radiation Protection of Staff During Interventional Cardiology. 2018 Available from: https://www.iaea.org/resources/rpop/health-professionals/interventionalprocedures/interventional-cardiology/staff#1..

[10] Goldburgh M Society for Imaging Informatics in Medicine. [Online]. 2017 Available from: http://siim.org/?page=archiving_chapter7 [cited 2017 September 7].

[11] Bellon E, Feron M, Deprez T, Reynders R, Van den Bosch B Trends in PACS architecture. Eur J Radiol 2011; 78: 199–204. doi: 10.1016/j.ejrad.2010.05.025 20566253

[12] Carter C, Veale B Digital Radiography and PACS. 2nd ed Missouri: Elsevier; 2013.

[13] The Royal College of Radiologists Picture archiving and communication systems (PACS) and guidelines on diagnostic display devices. 2nd edn London; 2012.

[14] Davies M REQUEST: Information about Siemens installed software/hardware In: Siemens Healthcare Limited: [e-mail]; 2017.

[15] ICRU Patient dosimetry for X rays used in medical imaging (Report 74). Journal of the ICRU 2005; 5. 10.1093/jicru/ndi016 24170883

[16] Kwon D, Little MP, Miller DL Reference air kerma and kerma-area product as estimators of peak skin dose for fluoroscopically guided interventions. Med Phys 2011; 38: 4196–204. doi: 10.1118/1.3590358 21859021 PMC3145218

[17] Huda W Kerma-area product in diagnostic radiology. AJR Am J Roentgenol 2014; 203: W565–9. doi: 10.2214/AJR.14.12513 25415721

[18] KKco Ltd Multipurpose Chest Phantom N1 "LUNGMAN". [Online]. 2017 Available from: https://www.kyotokagaku.com/products/detail03/ph-1.html [cited 2017 September 18].

[19] Leeds Test Objects Leeds Test Objects. [Online].. 2015 Available from: http://www.leedstestobjects.com/wp-content/uploads/TOR-18FG-product-specifications1.pdf. [cited 2017 September 4].

[20] Landis JR, Koch GG The measurement of observer agreement for categorical data. Biometrics 1977; 33: 159–74. doi: 10.2307/2529310 843571

[21] Shah A, Das P, Subkovas E, Buch AN, Rees M, Bellamy C Radiation dose during coronary angiogram: relation to body mass index. Heart Lung Circ 2015; 24: 21–5. doi: 10.1016/j.hlc.2014.05.018 25065542

[22] Dehen L, Vilmer C, Humilière C, Corcos T, Pentousis D, Ollivaud L, et al Chronic radiodermatitis following cardiac catheterisation: a report of two cases and a brief review of the literature. Heart 1999; 81: 308–12. doi: 10.1136/hrt.81.3.308 10026359 PMC1728981

[23] Liu F, Hernandez-Cabronero M, Sanchez V, Marcellin M, Bilgin A The current role of image compression standards in medical imaging. Information 2017; 8: 131. doi: 10.3390/info8040131 PMC8525863 34671488

